# Understanding the Better Than Average Effect on Altruism

**DOI:** 10.3389/fpsyg.2020.562846

**Published:** 2021-01-07

**Authors:** Yunyu Xiao, Kelly Wong, Qijin Cheng, Paul S. F. Yip

**Affiliations:** ^1^School of Social Work, Indiana University–Purdue University Indianapolis, Indianapolis, IN, United States; ^2^School of Social Work, Indiana University Bloomington, Bloomington, IN, United States; ^3^Centre for Suicide Research and Prevention, The University of Hong Kong, Pokfulam, Hong Kong; ^4^Department of Social Work, The Chinese University of Hong Kong, Shatin, Hong Kong; ^5^Department of Social Work and Social Administration, The University of Hong Kong, Pokfulam, Hong Kong

**Keywords:** Hong Kong, altruism, latent class analysis, prosocial behavior, better than average, altruistic behaviors

## Abstract

Prior research suggests that most people perceive themselves to be more altruistic than the average population, an observation known as the better-than-average (BTA) effect. Understanding the BTA effect carries significant public health implications, as self-perceived altruism is closely related to altruistic behaviors, which plays a significant role in individual and societal well-being. However, little is known about whether subpopulations with specific sociodemographic profiles are more likely to hold BTA altruistic self-perceptions, making it difficult to design targeted programs based on multiple sociodemographic characteristics to promote altruistic behaviors. This study addresses this gap by identifying the sociodemographic profiles of populations who are more likely to exhibit BTA effects on trait altruism. Data were derived from a representative sample of Hong Kong citizens (*n* = 1,185) in the 2017 Hong Kong Altruism Survey. A latent class analysis was performed using four domains of sociodemographic characteristics: sex, age, religion, and socioeconomic status. Multivariate multinomial logistic regressions were conducted to examine associations between class membership, BTA effect, and altruistic behaviors. The results yielded four classes of sociodemographic profiles. Middle-aged, Christian/Catholic, highly educated, and high-income individuals (Class 4, 17.8%) were most likely to exhibit BTA effects and behave altruistically; Class 3 (14.0%) were older, male, no/other religious belief, low education, and least likely to exhibit BTA effects and behave altruistically. Findings improve the understanding of the sociodemographic profiles of people showing BTA effects and facilitate targeted policy development to effectively promote altruism.

## Introduction

The enigmatic nature of altruism has intrigued researchers and philosophers over the centuries. Altruism is defined as helping behaviors purposefully to promote non-significant others’ welfare without expectation of external reward (Kerr et al., 2004; Wilson, 2015). Although the scope and measurements of altruism remain unstandardized and ambiguous, recent studies have consistently demonstrated a promising link between altruism and well-being (Kerr et al., 2004; Post, 2005; Killen and Macaskill, 2015; Au et al., 2020). Empirical studies have shown that altruism is associated with increased happiness (Borgonovi, 2008), physical health (Post, 2005), and life satisfaction (Dulin et al., 2001). Similar findings have been found cross-culturally, revealing the universal benefits of altruism (Feng and Guo, 2016). Therefore, the benefits associated with altruism call for a deeper understanding of the strategies required to promote altruism across populations effectively.

A promising line of research suggests that self-perceptions play a significant role in influencing altruistic behaviors, providing insight into a possible direction for promoting altruism (Lauren et al., 2017). Strengthening altruistic self-perceptions may reinforce altruistic behaviors (Xu et al., 2018). These findings suggest an interactive relationship between self-perceptions and behaviors, an observation consistent with the cognitive dissonance theory. According to the theory, when an individual holds inconsistent attitudes, beliefs, and behaviors, experiences of mental discomfort will lead to changes in any domain to restore balance and relieve discomfort (Bem, 1972; Lauren et al., 2017). Viewing oneself as an altruistic person might, therefore, motivate individuals to act in accordance with their self-perceptions, thereby activating a reinforcement process that induces future altruism (Lauren et al., 2017).

Previous research has identified the better-than-average (BTA) effect. Most people view themselves BTA across characteristics, ranging from driving skills (Roy and Liersch, 2013) to grammar ability (Kruger and Dunning, 1999), and to morality (Tappin and Mckay, 2017). Theoretical and motivational accounts often regard the BTA effect as a cognitive and judgment bias, as it is statistically impossible for most to be BTA across different dimensions (Kim et al., 2017). Theoretically, the “self-centrality breeds self-enhancement” principle postulates that people will perceive themselves BTA on traits that they deem to be more personally important (Sedikides and Strube, 1997; Sedikides et al., 2013). In particular, the motivational perspective of the BTA effect suggests that people are motivated to view themselves positively to maintain a positive, well-protected self-concept (Sedikides and Strube, 1997). Empirical support of the motivational argument of self-enhancement principle found the BTA effect to depend on trait self-importance, rather than character traits: agentic (competitive, ambitious) people viewed themselves better across agentic traits, whereas communal people viewed themselves better across communal traits (Gebauer et al., 2013).

Based on this motivational account, we hypothesize that most people will perceive themselves as more altruistic than the average population. Still, individuals who endorse more strong altruistic values will be more likely to exhibit the BTA effect on trait altruism. However, little is known about the population groups that hold different levels of altruistic self-perceptions and which population groups hold accurate versus inaccurate altruistic self-perceptions. Identifying the sociodemographic profiles of groups perceiving themselves BTA and their associations to actual altruistic behaviors showed great promises to promote altruism (Lauren et al., 2017). Since altruism not only benefits individuals but society (Cheng et al., 2017), it is possible that policies targeting specific groups with BTA can improve altruistic self-perceptions and actual altruistic behaviors more effectively, and thus enhancing the overall well-being of the societies.

### Characteristics That Affect Self-Centrality of Altruism

Many factors have the potential to impact how important altruism is to an individual. This section explored whether four common sociodemographic variables influence an individual’s likelihood of endorsing altruistic values to predict whether they perceive themselves BTA on trait altruism.

#### Sex

The social role theory states that the historical divide in social roles between males and females drove the rise of numerous sex stereotypes, all of which continue to impinge on people’s beliefs, identity, and reality (Eagly and Crowley, 1986). Previous evidence showed that females are more often associated with empathy and compassion, while males are often associated with competition and aggression (Belansky and Boggiano, 1994). Besides, when asked to predict another participants’ donation pattern in an experimental game, results found that participants had accurate perceptions of males’ average altruistic level, but tended to perceive females to be more altruistic than males, and also tended to overestimate females’ altruistic levels (Brañas-Garza et al., 2018). As the social identity theory posits that females are likely to adopt positive values and stereotypes that characterize their social group, this finding provides substantial evidence that identification as a female can facilitate more altruistic value systems (Tajfel, 1982; Czopp et al., 2015). This notion is supported by studies that suggest females hold stronger communal values and prioritize altruism significantly more than males (Dietz et al., 2002). As such, we chose to include sex as the first BTA-related sociodemographic profile composition and hypothesize that females will value altruism relatively more than males and thereby exhibit a stronger BTA effect on trait altruism.

#### Age

Brandtstädter et al. (2010) found that older adults behave more altruistically toward strangers than younger adults. The altruistic behavior toward strangers among older adults can be explained by the social-discounting framework (Jones and Rachlin, 2006). When performing altruistic behaviors to socially distant others (e.g., total strangers), people rely less on the reciprocal-altruism motive, but more on the ego-transcendent motive, given fewer possibilities for future reciprocation (Osiński, 2009). This provides evidence that older adults may have stronger ego-transcending motives, less likely to associate altruistic behaviors with personal interests, and thus share more generosity toward socially distant others such as total strangers (Carstensen, 1992; Brandtstädter et al., 2010; Hubbard et al., 2016). Similarly, large-scale, cross-cultural studies also found that older adults, compared to younger adults, were more concerned about the environment than their wealth and endorsed stronger communal rather than agentic values (Freund and Blanchard-Fields, 2014). We chose to include age group as the second BTA-related sociodemographic profile composition and hypothesize that older adults are more likely to endorse stronger altruistic values and thereby perceive themselves BTA on trait altruism (Killen and Macaskill, 2015; Fung et al., 2016).

#### Religion

As altruism is a core value in most of the world’s religions (Neusner and Chilton, 2005), we hypothesize that religious individuals may hold stronger altruistic values than non-religious individuals. In support of this hypothesis, Ji et al. (2006) studied the relationship between religious dimensions (extrinsic, intrinsic, doctrinal orthodoxy, and faith maturity) and altruism in protestant adolescents. Results found religiosity to be positively related to altruistic values, though intrinsic religion (religion as an end) and doctrinal orthodoxy were negatively associated with altruistic behaviors (Ji et al., 2006). Further support comes from a study that found that religious individuals exhibited stronger BTA effects for trait warmth (as a Christian stereotype), but not for competence (not a Christian stereotype). However, it is worth noting that most religions tested in the aforementioned studies share a degree of similarity with Christianity, meaning that BTA effects on trait altruism may vary across religions.

In Hong Kong, Christian/Catholic organizations frequently engage in a wide range of social issues. Examples include Christian Action that offers food assistance to needy populations, and Christian Concern for the Homeless Association that provides holistic support to homeless individuals in Hong Kong (Christian Concern for the Homeless Association, 2019; Christian Action Hong Kong, 2020). Given the unique role of religion in Hong Kong, we chose to include religious groups as the third dimension of BTA-related sociodemographic profile composition and hypothesize that religious individuals are more likely to endorse stronger altruistic values and exhibit a stronger BTA effect on trait altruism.

#### Socioeconomic Status

Socioeconomic status (SES) is commonly determined by educational attainment and income levels (Winkleby et al., 1992). It reflects an individuals’ relative social and economic standing and influences their choices, experiences, and values (Manstead, 2018). People with higher educational attainment may have a greater awareness of societal problems and are embedded in educational institutions/social groups, which may increase their positive norms in altruism (Bekkers, 2004). Education was also positively linked with self-transcendence (universalism/benevolence) values in most countries (Meuleman et al., 2012). Conversely, income had inconsistent effects on a range of values, including self-transcendence (Reyes-García et al., 2016).

However, other lines of research suggest that SES may also be negatively associated with altruism. Piff et al. (2010) found lower SES individuals to be more altruistic in a trust game than their higher SES counterparts. This may reflect social expectations for individuals from upper and lower SES, as temporarily manipulated, “low status” groups were also found to hold stronger self-transcendent values and goals related to enhancing others’ welfare/helping others, compared to “high status” groups (Guinote et al., 2015). Despite mixed findings, the higher-SES population may have more time investigating altruistic behaviors, which is a prevalent situation in Hong Kong. Thus, we chose to include SES, as conceptualized by educational and income levels, as the fourth element of the BTA-related sociodemographic profile composition and hypothesize that individuals with higher education and lower income will endorse stronger altruistic values, and therefore perceive themselves BTA on trait altruism.

### Present Study

While previous studies have well investigated the sociodemographic correlations with altruistic behaviors discussed above, most ignored the fact that these sociodemographic characteristics may coalesce to specific and meaningful patterns representing certain groups of subpopulations. In the current study, we aim to build a profile of the people who perceive themselves as more altruistic than the average population in Hong Kong, a Special Administrative Region of China, a modern metropolis with a 7.4 million population, ranked 15th in GDP (US$46,000) and 96% ethnically Chinese society (HKSAR Census and Statistics Department, 2015). The focus on Hong Kong allows us to focus on the specific cultural and societal background in a non-Western context. For example, societies with greater sex equality reduce differences in values and beliefs, weakening BTA effects (Schwartz and Rubel-Lifschitz, 2009). Similarly, societies with greater economic inequality strengthen the need to differentiate the self-upward from others, enhancing BTA effects (Loughnan et al., 2011). To this end, we used data from the Hong Kong Altruism Index Survey in 2017. We carried out a latent class analysis (LCA) to identify the distinct sociodemographic profiles and examined their associations with the BTA effect. The profile for BTA effects in Hong Kong would be of interest compared to the findings of Western countries as Hong Kong is still dominant by Chinese cultural value but with much western exposure. It is still experiencing sex inequality and high economic inequality (Chan and Cheng, 2012; Oxfam, 2018).

Based on this review, we hypothesize that females, middle-aged, Christians, highly educated, and lower-income individuals perceive the self to be more altruistic than average. Specifically, the rationale of choosing sex derived from previous findings suggesting males had more self-important perception than females (Gebauer et al., 2013), while females tended to be more altruistic and overestimate their altruistic levels than males (Brañas-Garza et al., 2018). We chose age as older adults were found to share more communal traits and altruistically than younger adults, possibly due to their stronger ego-transcending motives and fewer self-centered interests (Brandtstädter et al., 2010). Religion was chosen for consistent findings in prior studies showing people with religious beliefs report greater altruistic values and more likely to engage in actual altruistic behaviors (Neusner and Chilton, 2005; Ji et al., 2006). Last, we chose SES for its positive relationship with a wide range of altruistic behaviors (Chou et al., 2003).

In sum, our selection of variables was based on three main reasons: (a) First, Gebauer et al. (2013) found agentic traits to be more self-important to younger adults, non-religious individuals, males, and in agentic cultures, whereas communal traits were more self-important in older adults, religious individuals, females, and in communal cultures. The findings suggest that there are multiple sociodemographic characteristics that influence the values that an individual adopts, which will ultimately influence the exhibition of BTA effects. Our study adds to this by exploring how these sociodemographic variables together explain the BTA effect in altruistic self-perception. (b) Second, the purpose of the study is not to provide an exhaustive list of all the characteristics associated with the BTA effect on trait altruism. Instead, the study aims to find the different subgroups that display different patterns of BTA and corresponding altruistic behaviors, to facilitate policy development and interventions. With such purpose in mind, it is more appropriate to focus on the common sociodemographic characteristics that allow us to better identify and classify the Hong Kong population into different subgroups. (c) Third, previous literature in Hong Kong provided insight into the sociodemographic factors related to volunteering/altruistic behaviors. For instance, Chou et al. (2003) found that soon-to-be old adults who planned to be volunteers were more likely to have higher education and income, addressing the importance of understanding altruism through variables co-occurring together that forms the different subgroups like our study, rather than looking at the variables separately.

Our research offers several significant contributions to the existing literature and policymaking: (a) First, we adopted a novel methodological approach to building a profile of populations who view themselves as BTA on trait altruism. This extends the previous literature focusing on the predictors of the BTA effect. While those approaches inform us of the significant contribution each variable has in predicting the BTA effect, it does not inform the characteristics of people who hold stronger altruistic self-perceptions. (b) Second, self-perceived altruism is strongly correlated with altruistic behaviors. Building a profile of populations who view themselves more or less altruistic will give us insight into how we may devise more targeted interventions specific to different subpopulations to promote altruistic behaviors for population well-being effectively.

## Materials and Methods

### Data and Sample

The Hong Kong Altruism Index Survey was a 2-year territory-wide panel survey initiated by the Hong Kong Jockey Club Center for Suicide Research and Prevention, University of Hong Kong, which aimed to collect information on altruism well-being in Hong Kong residents. There were two waves of data collection for the surveys, with the first in 2016 and the second in 2017. In the first wave of study, Hong Kong residents aged 15 or above were randomly selected by interviewers from registered Hong Kong mobile numbers allocated by the Office of the Communications Authority, Government of HKSAR, from 6:30 pm to 10:30 pm. The interview was conducted by the Social Sciences Research Centre, The University of Hong Kong (SSRC), from August to November 2017. A total of 3,016 participants were successfully completed in the first-round interview, with 2,340 participants (77.6%) having agreed to participate in the second-round interview. In the second wave of the study, data were collected using the SSRC Computer-Assisted Telephone Interview system. Interviewers randomly dialed the mobile phone numbers among the 2,340 respondents who agreed to a second-round interview, from 6:30 pm to 10:30 pm. As soon as the telephone was connected, interviewers confirmed their identity by asking whether they were the subscriber or primary user of the mobile telephone number. A total of 1,185 participants (39.3%) had been successfully interviewed in the 2017 follow-up survey. This study was approved by the Human Research Ethics Committee for Non-Clinical Faculties of the University of Hong Kong (Research Ethics Approval ID: EA1605026). Further information about the survey report (hereafter “2017 Report”) is made available online: https://csrp.hku.hk/wp-content/uploads/2018/06/2017_Hong_Kong_Altruism_Index_Survey_CN.pdf.

The analytic sample in this study consisted of 1,185 individuals using the second-wave data in the 2017 Hong Kong Altruism Index Survey, which excluded individuals who had missing information on all five questions of sociodemographic characteristics. We focused on the second-wave data to provide more updated information on profiles of BTA among the Hong Kong population. Sociodemographic characteristics of the study sample and comparison with the 2016 baseline sample are presented in Appendix 1. There were no statistical differences in sex and age between 2016 and 2017. No significant differences in actual altruistic behavior indices were found, except for informal altruistic behaviors (*p* = 0.011). Further analyses showed few sociodemographic differences associated with scores of altruistic behaviors in the second wave in comparison with the first wave. For example, sex was not associated with the changes in any altruistic behaviors between 2016 and 2017 (Appendix 2).

### Measures

#### Sociodemographic Characteristics

Five items in the 2017 Hong Kong Altruism Index survey were recoded to encompass four dimensions of sociodemographic characteristics: sex, age, religious beliefs, and SES (education and household income). *Sex* was measured by self-reported biological sex (female, male). *Age* was categorized into four groups (15–34, 35–54, 55–64, and ≥65 years old). *Religious belief* was recoded into four categories (no religion, Christian/Catholic, Buddhism, and other). *Educational level* was measured by three levels, including post-secondary or above, secondary school, and primary school or below. *Household income* was categorized into lower than $12,999, $13,000–$24,999, $25,000–$79,999, and over $80,000.

#### Better-Than-Average Effect

The status of BTA was constructed using two questions concerning respondents’ self-perceived altruistic level and perception of overall Hong Kong people’s altruistic level. Individuals were asked to rate “How helpful do you think you are?” and “How willing do you think people in Hong Kong would help each other out in general?” using a seven-point scale (1 = absolutely not willing, 7 = absolutely willing in any circumstances). People who had higher rates in self-perceived altruistic level than the level of their perception of overall Hong Kong people were considered BTA.

#### Covariates: Actual Altruistic Behaviors

Four sub-dimensions of the Hong Kong Altruism Index (“A-Index”), namely, formal volunteering, formal monetary donation, blood and organ donation, and informal helping, were included to measure individuals’ actual altruistic behaviors. The A-Index was grounded in theory, adjusted for the optimal time frame setting for 10 altruistic or prosocial behaviors, and previously validated to reflect organic and stable traits of altruism (Cheng et al., 2017). The score ranges are formal volunteering (0–2), formal monetary donation (0–1), blood and organ donation (0–2), and informal helping (0–5).

#### Altruism Value

Altruism value was measured concerning whether respondents viewed altruism as a source of happiness. Individuals were asked to rate, “To what extent do you agree: Helping others makes you happy” using a five-point scale (1 = lowest; 5 = highest). Individuals who agreed to this statement more were considered to value altruism more.

### Statistical Analysis

#### Descriptive Statistics

Descriptive statistics for sociodemographic characteristics, self-perceived altruism, and actual altruistic behaviors of the study sample and comparisons across groups were assessed using Stata version 16.0 (Stata Corp., College Station, TX, United States). To ensure the accuracy of model estimates, sampling weights were applied to adjust for the sampling design. To improve the representativeness of the study, data have been weighted by sex, age, education level, and economic status of the Hong Kong population, as presented in the General Household Survey Census and Statistics Department (HKSAR Census and Statistics Department, 2015).

#### Correlational Analysis

Kendall’s tau correlation analysis was applied to examine the relationship between altruism value and the BTA effect. Statistical significance was taken at a *p* < 0.05 level.

#### Latent Class Analysis

Latent class analysis was applied to investigate the heterogeneity in sociodemographic characteristics among the whole population and identify possible, empirically defined, and meaningful subgroups. LCA is a well-validated, person-centered statistical technique that uses mixture modeling to examine the best-fitting model for a set of data. The central hypothesis of LCA, which is an inherently iterative process, is that a heterogeneous population can be reduced to several homogeneous and unobserved groups or classes through assessing and minimizing the associations in responses across multiple indicator variables (Hagenaars and Mccutcheon, 2002; Vermunt and Magidson, 2002; Vermunt, 2010). LCA has been widely used in psychology, psychiatry, public health, and other medical disciplines (Jung and Wickrama, 2008; Calfee et al., 2014; Xiao et al., 2019). Contrary to the traditional regression analysis, in which the goal is to understand the association between pre-defined independent variables and target outcome, LCA does not mandate a known outcome but asks whether there are subpopulations defined by a combination of variables (Calfee et al., 2014). LCA also differs from the traditional cluster analysis in that it is person-centered and model-based, whereas the standard clustering is a variable-centered approach that employs probability-based classification algorithms that cluster cases on predetermined criteria, a model−based approach utilizes probability−based classification (Calfee et al., 2014).

We utilized a three-step approach so that the measurement model remained fixed when introducing the covariates (Vermunt, 2010; Asparouhov and Muthén, 2014). Each sociodemographic variable was treated as categorical variables. Since the exact number of latent classes representing the sociodemographic subgroups were unknown, an exploratory approach was used, which started with the most parsimonious one-class model and assessed successive models through increasing the numbers of classes. Each latent class solution was replicated 20 times with random starting values and 1,000 iterations. This method included a close examination of item loadings and model fit indices for estimated latent classes (Vermunt, 2010; Asparouhov and Muthén, 2014).

To determine the final number of latent classes, we considered conceptual meaning, entropy (Asparouhov and Muthén, 2014), smallest estimated class proportions (Nylund et al., 2007), and several statistical model fit indices (Nylund et al., 2007), such as the Akaike information criterion (AIC) and adjusted Bayesian information criterion (BIC). Latent classes with less than 5% of the total sample were excluded due to the possibility of poor generalizability (Finch and Bolin, 2017) and overextraction in the presence of non-normal data (Bauer and Curran, 2003). Mplus accounted for the sampling design by correcting the standard errors and Chi-square tests of model fit (Muthén and Muthén, 2015). Maximum likelihood estimation with robust standard errors incorporating all available data was used to deal with missing data and to estimate parameters.

#### Multivariate Multinomial Logistic Regression and Multivariate Logistic Regression

Following the identification of the appropriate number of latent classes, a series of cross-tabulations were conducted to examine the distribution of classes across BTA and actual altruistic behaviors. *Chi-square* tests and analysis of variance (ANOVA) were conducted to examine bivariate associations. Multinomial logistic regression was conducted to investigate the associations between class membership, BTA, and four types of altruistic behaviors (Asparouhov and Muthén, 2014). Different reference groups were used to allow various group comparisons. Last, multivariate logistic regressions were performed using the identified classes to predict the likelihood of being BTA, controlling for the actual altruistic behaviors. Statistical significance was taken as a two-sided *p* < 0.05.

## Results

### Correlation Between Altruism Value and the Better-Than-Average Effect (BTA)

Using Kendall’s tau correlation analysis, results found that altruism values had a positive but weak correlation with the BTA effect (τ = 0.108, *p* < 0.001).

### Latent Class Models of Sociodemographic Characteristics

Results of the estimation of latent class models from one- to six-group solutions were examined since the best log-likelihood value of the seven-class model was not replicated (Table 1). Across the six models, the AIC decreased, while the four-class model had the lowest adjusted BIC. The entropy of the four-class model (0.704) was beyond the criteria for good class separation (i.e., entropy = 0.60; Asparouhov and Muthén, 2014). Given that the four-class solution also provided the most conceptually coherent description of sociodemographic profile and contained reasonable sample size distribution of class (smallest class has over 5%; Finch and Bolin, 2017), it is chosen as the most appropriate solution.

**TABLE 1:** Summary of latent class model identification and fit statistics.

No. of classes	AIC	Adjusted BIC	Smallest class, %	Entropy
1	22,603.739	22,641.762		
2	11,610.497	11,658.025	31.32%	0.772
3	11,537.764	11,610.007	19.15%	0.670
**4**	**11,501.798**	**11,598.756**	**14.01%**	**0.704**
5	11,481.415	11,603.087	11.70%	0.668
6	11,473.124	11,619.511	5.49%	0.696

*AIC, Akaike information criterion; BIC, Bayesian information criterion. Bolded row represents the identified model.*

Table 2 shows the estimated item probabilities for the four identified sociodemographic profiles. Class 1 (18.89%) had the highest probabilities of being female, middle, and old age (55–64 years old), Buddhism, and having a low income (≤$12,999). Class 2 (49.27%) is most likely to be young and middle-aged (15–54 years old), no religious belief, having a secondary-school level of education, and low-middle income ($13,000–$79,999 HKD) individuals. Class 3 (14.01%) had the highest probabilities of being male, older adults (≥65 years old), no or other religious beliefs, and having primary or lower educational levels. Class 4 (17.84%) had middle-aged individuals (35–54 years old), Christian/Catholic, having post-secondary or above educational level, and high-income (≥$80,000).

**TABLE 2:**
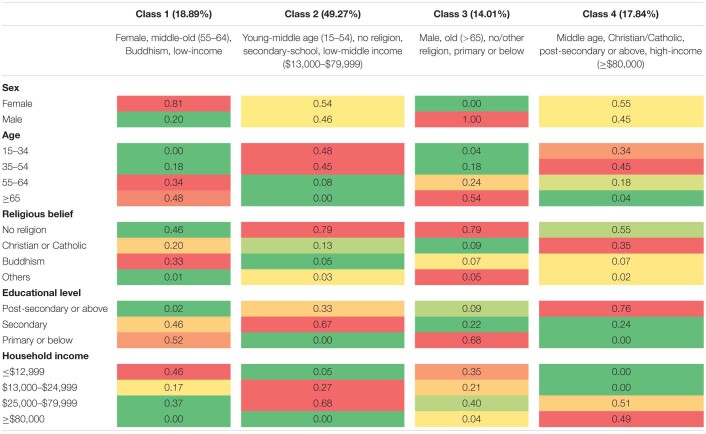
Four-class model: Estimated probabilities by latent class membership.

*LCA, latent class analysis adjusted for sampling weights. Red indicates high probabilities, green indicates low probabilities, and yellow indicates probabilities in between. Percentages of classes are weighted.*

### Distribution of Better-Than-Average (BTA) and Actual Altruistic Behaviors by Class Models

Table 3 shows that individuals in Class 4 (*middle age, Christian/Catholic, post-secondary or above, high income*) had the highest rates of being BTA (83.97%) across all four subgroups (*p* < 0.001). In addition, Class 4 was most likely to participate in formal volunteering [mean (*M*) = 0.91, standard deviation (*SD*) = 0.83], formal monetary donation (*M* = 0.90, *SD* = 0.30), and blood and organ donation (*M* = 1.04, *SD* = 0.75) compared to other classes (*p* < 0.001). Contrasting with this, individuals in Class 3 (*male, old, no/other religion, primary or below*) had the lowest participation in formal volunteering (*M* = 0.46, *SD* = 0.68) and formal monetary donation (*M* = 0.69, *SD* = 0.47). Class 1 (*female, middle-old, Buddhism, low income*) had the lowest rate of donating blood or organs (*M* = 0.45, *SD* = 0.67).

**TABLE 3:** Descriptive statistics of better-than-average (BTA) and altruistic behaviors by sociodemographic latent classes.

	Sociodemographic profile				
	Class 1	Class 2	Class 3	Class 4	*P*
BTA and actual altruistic behaviors	Female, middle-old (55–64), Buddhism, low-income	Young-middle age (15–54), no religion, secondary-school, low-middle income ($13,000–$79,999)	Male, old (>65), no/other religion, primary or below	Middle age, Christian/Catholic, post-secondary or above, high-income (≥$80,000)	
	(*n* = 224, 18.89%)	(*n* = 584, 49.27%)	(*n* = 166, 14.01%)	(*n* = 211, 17.84%)	

**BTA**					<0.001*^*a*^*
No	26.97	21.93	33.82	16.03	
Yes	73.03	78.07	66.18	83.97	
**Altruistic behaviors**					
Formal volunteering	0.60 (0.75)	0.77 (0.82)	0.46 (0.68)	0.91 (0.83)	<0.001*^*b*^*
Formal monetary donation	0.87 (0.34)	0.83 (0.37)	0.69 (0.47)	0.90 (0.30)	<0.001*^*b*^*
Blood and organ donation	0.45 (0.67)	0.83 (0.75)	0.51 (0.61)	1.04 (0.75)	<0.001*^*b*^*
Informal helping	2.85 (1.36)	2.94 (1.15)	2.69 (1.35)	3.07 (1.13)	0.067

*^a^Chi-square (χ^2^). ^b^ANOVA.*

### Sociodemographic Profiles of Better-Than-Average (BTA)

Using multinomial logistic regression results in Table 4 shows that compared to Class 1, individuals in Class 4 were 2.41 times (95% CI = 2.25–2.58) more likely to be BTA. This means individuals in Class 4 had 141% greater odds to be BTA than those in Class 1 indicating that individuals in Class 4 had 155% greater odds to have BTA perception than those in Class 3. Similarly, Class 4 individuals were 2.55 times (95% CI = 2.37–2.74) more likely to be BTA than people in Class 3. This result was held consistent even when controlling for actual altruistic behaviors (Table 5), where people in Class 4 had 2.22 more odds (95% CI = 1.12–4.38) of exhibiting BTA than those in Class 3.

**TABLE 4:** Multinomial regression of better-than-average (BTA) and altruistic behaviors by sociodemographic latent classes.

	**Class 2**		**Class 3**		**Class 4**	
	
BTA and actual altruistic behaviors (Reference Group: Class 1)	Young-middle age (15–54), no religion, secondary-school, low-middle income ($13,000–$79,999)		Male, old (>65), no/other religion, primary or below		Middle age, Christian/Catholic, post-secondary or above, high-income (≥$80,000)	
	
	OR (95% CI)	*p*	OR (95% CI)	*p*	OR (95% CI)	*p*

BTA	1.83 (0.84–4)	0.13	0.94 (0.43–2.05)	0.92	**2.41 (1.11–5.24)**	**0.04**
**Altruistic behaviors**						
Formal volunteering	1.29 (0.59–2.8)	0.25	0.71 (0.33–1.54)	0.34	1.52 (0.7–3.31)	0.06
Formal monetary donation	**0.34 (0.16–0.74)**	**0.06**	**0.12 (0.06–0.27)**	**0.00**	0.72 (0.33–1.56)	0.60
Blood and organ donation	**3.57 (1.64–7.78)**	**0.00**	1.86 (0.86–4.06)	0.18	**5.23 (2.4–11.39)**	**0.00**
Informal helping	1.1 (0.5–2.39)	0.58	1.08 (0.5–2.36)	0.74	1.13 (0.52–2.46)	0.48

	Class 1		Class 3		Class 4	
BTA and actual altruistic behaviors (Reference Group: Class 2)	Female, middle-old (55–64), Buddhism, low-income		Male, old (>65), no/other religion, primary or below		Middle age, Christian/Catholic, post-secondary or above, high-income (≥$80,000)	
	
	OR (95% CI)	*p*	OR (95% CI)	*p*	OR (95% CI)	*p*

BTA	0.54 (0.25–1.19)	0.13	0.51 (0.24–1.12)	0.10	1.31 (0.6–2.86)	0.36
**Altruistic behaviors**						
Formal volunteering	0.78 (0.36–1.69)	0.25	**0.55 (0.25–1.2)**	**0.03**	1.18 (0.54–2.57)	0.23
Formal monetary donation	2.96 (1.36–6.44)	0.06	**0.37 (0.17–0.8)**	**0.01**	**2.12 (0.97–4.62)**	**0.04**
Blood and organ donation	**0.28 (0.13–0.61)**	**0.00**	**0.52 (0.24–1.14)**	**0.02**	**1.47 (0.67–3.19)**	**0.01**
Informal helping	0.91 (0.42–1.98)	0.58	0.99 (0.45–2.15)	0.95	1.03 (0.47–2.24)	0.73

BTA and actual	Class 1		Class 2		Class 4	
altruistic behaviors (Reference Group: Class 3)	Female, middle-old (55–64), Buddhism, low-income		Young-middle age (15–54), no religion, secondary-school, low-middle income ($13,000–$79,999)		Middle age, Christian/Catholic, post-secondary or above, high-income (≥$80,000)	
	
	OR (95% CI)	*p*	OR (95% CI)	*p*	OR (95% CI)	*p*

BTA	1.06 (0.49–2.31)	0.92	1.94 (0.89–4.23)	0.10	**2.55 (1.17–5.55)**	**0.04**
**Altruistic behaviors**						
Formal volunteering	1.41 (0.65–3.07)	0.34	**1.82 (0.84–3.96)**	**0.03**	**2.14 (0.98–4.67)**	**0.01**
Formal monetary donation	**8.03 (3.69–17.48)**	**0.00**	**2.72 (1.25–5.91)**	**0.01**	**5.76 (2.65–12.54)**	**0.00**
Blood and organ donation	**0.54 (0.25–1.17)**	**0.18**	**1.92 (0.88–4.18)**	**0.02**	**2.81 (1.29–6.12)**	**0.00**
Informal helping	0.92 (0.42–2.01)	0.74	1.01 (0.46–2.2)	0.95	1.04 (0.48–2.27)	0.82

BTA and actual	Class 1		Class 2		Class 3	
altruistic behaviors (Reference Group: Class 4)	Female, middle-old (55–64), Buddhism, low-income		Young-middle age (15–54), no religion, secondary-school, low-middle income ($13,000–$79,999)		Male, old (>65), no/other religion, primary or below	
	
	OR (95% CI)	*p*	OR (95% CI)	*p*	OR (95% CI)	**p**

BTA	0.42 (0.19–0.9)	0.04	0.3 (0.35–1.66)	0.36	**0.45 (0.18–0.85)**	**0.04**
**Altruistic behaviors**						
Formal volunteering	0.22 (0.3–1.43)	0.06	**0.14 (0.39–1.85)**	**0.23**	**0.29 (0.21–1.02)**	**0.01**
Formal monetary donation	**0.63 (0.64–3.03)**	**0.60**	**0.37 (0.22–1.03)**	**0.04**	**0.48 (0.08–0.38)**	**0.00**
Blood and organ donation	**0.34 (0.09–0.42)**	**0.00**	**0.14 (0.31–1.49)**	**0.01**	**0.30 (0.16–0.77)**	**0.00**
Informal helping	0.17 (0.41–1.93)	0.48	0.09 (0.45–2.12)	0.73	0.18 (0.44–2.09)	0.82

*OR, odds ratio; 95% CI, 95% confidence interval. Bold rows indicate statistically significant mean differences at significance level α = 0.05.*

**TABLE 5:** Model estimates predicting membership in better-than-average (BTA) profiles.

	Better than average			
	OR	*p*	95%	CI
**Class (Ref: Class 3)**				
Class 1	1.08	0.85	0.49	2.38
Class 2	1.73	0.08	0.94	3.18
Class 4	2.22	0.02	1.12	4.38
**Altruistic behaviors**				
Formal volunteering	1.20	0.14	0.94	1.53
Formal monetary donation	1.40	0.17	0.87	2.27
Blood and organ donation	1.27	0.05	1.00	1.61
Informal helping	1.39	0.00	1.17	1.64
*Constant*	0.45	0.04	0.22	0.95

*OR, odds ratio; 95% CI, confidence interval. Class 1: female, middle-old (55–64), Buddhism, low-income; Class 2: young-middle age (15–54), no religion, secondary-school, low-middle income ($13,000–$79,999); Class 3: male, old (>65), no/other religion, primary or below; Class 4: middle age, Christian/Catholic, post-secondary or above, high-income (≥$80,000).*

## Discussion and Conclusion

To our knowledge, this study is the first to systematically identify the profiles of people who are more likely to exhibit BTA effects. Using LCA, we identified four types of sociodemographic profiles that are distinctively linked to the likelihood of viewing oneself as BTA on trait altruism. In general, the four classes all perceived themselves BTA on trait altruism. Specifically, findings suggested that individuals in Class 4 were more likely to hold BTA altruistic self-perceptions and comprise those in their middle age, Christian/Catholic, well-educated (post-secondary and above), and having a high income. They also participated in more formal volunteering, monetary donation, and blood/organ donation. On the contrary, Class 3 populations were least likely to hold BTA self-perceptions and were comprised of older males with low educational levels and no/other religious beliefs. These results have policy implications toward mechanisms that improve individual and societal well-being by encouraging people with specific sociodemographic profiles to engage in altruistic behaviors.

The finding that shows Class 4 populations to be more likely to display stronger BTA effects is supported by the vast literature, which suggests this group to endorse stronger altruistic values. It is also consistent with the previous conceptualization of the BTA effect (Kim et al., 2017), which posits that people who exhibit stronger BTA effects may accurately perceive their actual altruistic behaviors. Earlier studies also proposed that while skilled performers are not always accurate in self-perceptions, they are generally less likely to greatly under- or overestimate their performance than less skilled persons (Ehrlinger et al., 2008). Besides, the Class 4 population profile corresponds to Chou et al. (2003)’s results that soon-to-be old adults who planned to be volunteers in Hong Kong were more likely to have higher education and income. This could also be because Class 4 populations might have accumulated greater social capital and more opportunities to participate in altruistic activities (Veenstra, 2000; Lindström et al., 2001; Ajrouch et al., 2005).

On the other hand, Class 4 populations may reflect specific social or personal norms, which increases their awareness of social responsibilities and corresponding self-perceived altruism (Batson and Powell, 2003). Furthermore, the finding that Class 4 members are more likely to practice formal altruistic behaviors consolidates previous research showing prosocial behaviors are essential for building reputation and self-evaluative emotions (Kraus and Callaghan, 2016). In particular, Class 4 members may hold stronger self-enhancing (contrast to ego-transcending) motives to participate in formal help, as formal help is better organized and recorded, and can reinforce or enhance their social identity and social status. Additionally, with higher SES, individuals in Class 4 may have more autonomy in controlling their time and decide to participate more in volunteering behaviors. On the contrary, Class 3 populations, comprised of older males who are non-religious and have low SES, might have fewer opportunities and resources to practice altruistic behaviors (Ajrouch et al., 2005). Besides, since altruism is strongly emphasized across religious beliefs and religiousness is a strong predictor of prosocial behaviors (Midlarsky et al., 2012), lack of religious belief could reduce the likelihood of perceiving oneself as more helpful than the others. Furthermore, previous studies show inconsistent results of the association between SES and altruism (Piff et al., 2010; Guinote et al., 2015).

Our results delineate the effect of income and education (Meuleman et al., 2012), highlighting that low-income people, combined with other characteristics in Class 3, were less likely to exhibit BTA effects. Class 1 populations comprised of the middle and old-aged females, Buddhist, and low-income group are also less likely to be the BTA, which contrasts a previous study where BTA effects on the self-sacrificing dimension were consistent across age groups (Zell and Alicke, 2011; Pornpattananangkul et al., 2017). However, stronger altruistic values may not necessarily strengthen BTA effects in older adults, as ego-transcending motives contradict self-enhancement (BTA effect) motives—altruism in the former focuses on genuine concern for others; altruism in the latter focuses on enhancing/maintaining positive self-perceptions. Older adults may be driven by ego-transcending rather than self-enhancing motives or be more prone to act out the altruistic behaviors but not the intention (Zell and Alicke, 2011) and, thus, showing less BTA effects for self-sacrifice for middle-aged participants.

### Future Directions

Our findings help identify the sociodemographic profiles to target policy development and improve the effectiveness of programs to enhance altruism. For instance, altruism policies should differ for Class 3 and 4 populations. For Class 4 populations, policies should strengthen and increase adherence to altruistic values, self-perceptions, and behaviors. Increasing altruism nudges (e.g., making altruism norms salient) and fostering habits of personal reflection on altruism (e.g., questions on the importance/consequences of altruism) are directions that may strengthen the enforcement mechanism between altruistic self-perception and behaviors (Farsides et al., 2013; Capraro et al., 2019). While applicable to Class 3 populations, increasing accessibility and engagement to altruistic behaviors is the foremost priority for this population and may be achieved by targeting the interests/preferences for altruism within this population profile. For instance, males have demonstrated a stronger preference to volunteer in roles that place them in risky/authoritative situations than their female counterparts, with retired males more attracted by high-skill volunteer tasks (Mjelde-Mossey and Chi, 2005; Wymer, 2011). Among older adults, a previous gratitude intervention has also been shown to improve well-being significantly (Killen and Macaskill, 2015). Similarly, low SES individuals may also be more attracted by volunteering opportunities that target asset building (e.g., employment skills) and professional networks, as self-interest and altruistic reasons can both promote altruistic behaviors, forming the basis for activating and strengthening altruistic values (Weisinger et al., 2016; De Dominicis et al., 2017). Yet, further research is required to improve our understanding of different populations’ helping preferences, barriers, and expectations to more effectively develop and implement future altruism promotion policies (Mjelde-Mossey and Chi, 2005).

Furthermore, our results suggest that people who hold BTA altruistic self-perceptions are more likely to behave altruistically. People with BTA may perceive the tasks of acting altruistic behaviors as easy given their ability (Kim et al., 2017), and they are financially prepared and emotionally motivated to engage in actual altruistic behaviors. For instance, Class 4 is more educated with high-income status, allowing their choices to formally particulate in volunteering, suggesting that altruistic self-perceptions are important in predicting altruistic behaviors and shed light on policymakers’ potential direction in promoting altruistic behaviors. It supports belief–behavior consistency, emphasizing individuals’ desire to reach consistency between their beliefs/values and behaviors (Bem, 1972; Lauren et al., 2017). This aligns with, and is supported by, a study that tested the impact of moral nudges on cheating behavior, finding that people who signed honesty forms are less likely to cheat than those who did not sign honesty forms. This also addresses the importance of activating and strengthening BTA self-perceptions for populations; most importantly, Class 3 may need more strengthening, while Class 4 may require more activating self-perceptions.

### Strengths and Limitations

First, the strength of this study includes the use of LCA to identify the underlying subgroups of sociodemographic profiles that were more likely to be BTA. This approach can help to address methodological challenges that arise in subgroup analysis, including high rates of Type I error, restrictions of higher-order interactions, and low statistical power (Lanza and Rhoades, 2013). LCA can also facilitate the design of tailored interventions that allocate resources to subgroups and maximize intervention outcomes. The sampling strategy and large sample size for conducting LCA also strengthened the statistical power of analysis. Second, our study addressed diversity and inclusion by considering the various sociodemographic characteristics linked to the BTA effect and altruism. We further discussed the strategies to encourage altruistic behaviors across sex, age, religious belief, and SES groups. Third, this study was also backed by theoretical support. Although our results yielded a significant but weak association between altruism value and the BTA effect, this could be because our measure of altruism value was based on whether altruism was a source of happiness, without explicitly measuring how much respondents endorsed altruistic values to test the self-centrality breeds self-enhancement principle. However, we overcame this potential limitation by using a data-driven approach to identify four broad sociodemographic variables that have the most potential to influence our value systems with strong literature support (Gebauer et al., 2013). Therefore, because our variable selection and findings are theoretically supported, it provides stronger evidence that the self-importance of altruism influences enhanced self-perception.

Several limitations should be addressed in this study. First, our study employed an indirect measure of the BTA effect by asking participants to rate themselves and others on two separate scales. However, because direct measures (participants rate the self-compared to others on one scale) provide a stronger comparative frame for self-other differentiation, it has the potential to capture stronger BTA effects than indirect measures (Alicke and Govorun, 2013). Thus, we may have suppressed and underestimated the BTA effect in our participants. Future studies should consider employing both measures to ensure consistency and accuracy of our BTA effect measurement. Second, this study relied on self-report methods, with no objective measure of respondents’ actual altruism. Responses could possibly be distorted by social desirability bias, the tendency for respondents to respond in a manner that is deemed favorable. As in a previous study, the revealed 65.3% of their respondents to over-report their charitable giving is highly probable, exceeding 22.4% of the respondents that under-report their giving (Lee and Sargeant, 2011). This is especially relevant to Class 4 populations, which may be more motivated to exaggerate their helpfulness to match their self-perceptions. Therefore, future studies should consider controlling for social desirability bias to obtain more accurate altruism measures to achieve a greater understanding of the relationship between altruistic self-perceptions and behaviors. Third, our measures of actual altruistic behaviors focus more generally on helping or prosocial behaviors, without specifically asking if the participants perceive the helping behaviors as altruistic or not. It is noteworthy that the four dimensions in the A-index of the altruism measures in this study have shown good structural, theoretical, dimensional, and constructed validity (Cheng et al., 2017). Nevertheless, it is possible that individuals interpret altruism as helping oneself more than helping others, although the outcome will benefit others. For instance, a previous validation study indicated blood and organ donation might be affected by traits other than altruism (Cheng et al., 2017). Fourth, due to the data availability, only one question (“helping others makes you happy”) was used to serve as the proxy measure of “altruism value,” which may associate with low reliability or validity. However, previous studies have used this to indicate the meaningfulness of altruistic behaviors and were found to have a good psychometric property (Van Vuuren and Pothof, 2019). Future studies shall try incorporating multiple items to measure a scale of altruistic value. Last, this study used a cross-sectional design, which does not allow us to capture the change in sociodemographic profiles of populations who view themselves BTA over time. Yet, it forms the basis for future studies to track changes in BTA effects in these profiles over time or after policy implementations, and track whether their current levels of altruistic self-perceptions across all four classes predict future altruism.

## Data Availability Statement

The raw data supporting the conclusions of this article will be made available by the authors, without undue reservation.

## Ethics Statement

The studies involving human participants were reviewed and approved by Human Research Ethics Committee for Non-Clinical Faculties of the University of Hong Kong (Research Ethics Approval ID: EA1605026). The patients/participants provided their written informed consent to participate in this study.

## Author Contributions

YX conceptualized the study, contributed to the methodology, software, and formal analysis, wrote and prepared the original draft, wrote, reviewed, and edited the manuscript, and provided equal contribution. KW conceptualized the study, wrote and prepared the original draft, wrote, reviewed, and edited the manuscript, and provided equal contribution. QC wrote, reviewed, and edited the manuscript. PY conceptualized and supervised the study, acquired the funding, and wrote, reviewed, and edited the manuscript. All authors contributed to the article and approved the submitted version.

## Conflict of Interest

The authors declare that the research was conducted in the absence of any commercial or financial relationships that could be construed as a potential conflict of interest.

## Appendix 1

**TABLE 1:** Sociodemographic characteristics.

Sociodemographic characteristics	*2017*		*2016*		*p*^*a*^
	*n*	%	*n*	%	
**Sex**					
Female	560	47.26	1,481	49.10	0.281
Male	625	52.74	1,535	50.90	
**Age**					
15–34	511	43.12	1,280	42.44	0.940
35–54	441	37.22	1,136	37.67	
55–64	136	11.48	339	11.24	
≥65	97	8.19	261	8.65	
**Religious belief**					
No religion	821	69.28	1,920	63.98	0.001
Christian or Catholic	243	20.51	656	21.86	
Buddhism	93	7.85	290	9.66	
Others	28	2.36	135	4.50	
**Educational level**					
Post-secondary or above	665	56.12	1,571	52.09	0.013
Secondary	461	38.9	1,235	40.95	
Primary or below	59	4.98	210	6.96	
**Household income**					
≤$12,999	94	8.31	379	14.41	<0.001
13,000–$24,999	182	16.09	506	19.24	
25,000–$79,999	664	58.71	1336	50.78	
≥$80,000	191	16.89	410	15.58	
***Altruistic behaviors***	*M*	*SD*	*M*	*SD*	*p*^*b*^
Formal volunteering (0–2)	0.68	0.80	0.72	0.79	0.076
Formal monetary donation (0–1)	0.83	0.37	0.85	0.36	0.314
Blood and organ donation (0–2)	0.70	0.74	0.66	0.74	0.042
Informal helping (0–5)	2.90	1.23	3.01	1.23	0.011

*M, mean; SD, standard deviation. ^a^Bivariate comparisons using Chi-square (χ^2^). ^b^Bivariate comparisons using t-test.*

## Appendix 2

**TABLE 2:** Sociodemographic differences in changes in altruistic behavior between 2016 and 2017.^*a*^

	Formal volunteering					Formal monetary donation					Blood and organ donation					Informal helping				
	2016		2017		p	2016		2017		p	2016		2017		p	2016		2017		p
	M	SD	M	SD		M	SD	M	SD		M	SD	M	SD		M	SD	M	SD	
**Sex**																				
Female	0.79	0.80	0.75	0.816	0.136	0.88	0.33	0.89	0.319	0.686	0.62	0.75	0.67	0.765	0.093	3.16	1.2	3.05	1.22	0.051
Male	0.64	0.77	0.60	0.77	0.362	0.82	0.38	0.78	0.416	0.088	0.70	0.72	0.73	0.72	0.251	2.86	1.25	2.75	1.23	0.115
**Age**																				
15–34	1.13	0.58	0.90	0.82	<0.001	0.85	0.36	0.81	0.39	0.660	0.74	0.73	0.79	0.75	0.021	3.11	1.11	2.94	1.09	<0.001
35–54	1.13	0.55	0.70	0.81	<0.001	0.91	0.29	0.89	0.31	0.016	0.88	0.77	1.00	0.77	0.512	3.24	1.18	3.05	1.17	<0.001
55–64	1.04	0.54	0.70	0.81	0.003	0.82	0.38	0.87	0.34	0.562	0.68	0.72	0.83	0.72	0.109	2.86	1.29	2.84	1.31	0.741
≥65	0.96	0.52	0.53	0.71	0.184	0.82	0.39	0.79	0.41	0.495	0.46	0.63	0.41	0.59	<0.001	2.73	1.34	2.78	1.42	0.134
**Religious belief**																				
No religion	1.14	0.55	0.71	0.81	<0.001	0.85	0.35	0.82	0.38	0.810	0.73	0.74	0.82	0.76	0.001	3.00	1.18	2.89	1.17	0.046
Christian or Catholic	1.05	0.59	0.93	0.81	0.003	0.89	0.31	0.89	0.31	0.046	0.93	0.78	0.99	0.75	0.101	3.28	1.17	3.13	1.16	0.006
Buddhism	1.05	0.59	0.80	0.79	<0.001	0.87	0.33	0.94	0.25	0.519	0.61	0.68	0.66	0.71	0.029	3.33	1.18	3.00	1.25	0.002
Others	1.05	0.61	1.07	0.86	0.397	0.87	0.33	0.86	0.36	0.361	0.59	0.72	0.71	0.71	0.140	3.21	1.14	3.18	1.31	0.078
**Educational level**																				
≥Post-secondary	1.13	0.60	0.85	0.82	<0.001	0.89	0.31	0.86	0.35	0.007	0.92	0.75	0.99	0.75	0.008	3.19	1.14	2.99	1.16	<0.001
Secondary	1.11	0.52	0.71	0.81	<0.001	0.84	0.36	0.84	0.37	0.544	0.62	0.72	0.69	0.73	<0.001	3.07	1.20	2.94	1.17	0.052
≤Primary or below	0.96	0.50	0.37	0.67	0.046	0.80	0.40	0.73	0.45	0.376	0.34	0.60	0.31	0.53	<0.001	2.61	1.34	2.63	1.36	0.215
**Household income**																				
≤$12,999	1.03	0.50	0.54	0.74	0.010	0.78	0.41	0.77	0.43	0.430	0.48	0.68	0.49	0.70	<0.001	2.87	1.31	2.68	1.35	0.819
13,000–$24,999	1.12	0.53	0.77	0.80	<0.001	0.84	0.37	0.79	0.41	0.890	0.59	0.68	0.70	0.74	0.001	3.07	1.19	2.90	1.16	0.600
25,000–$79,999	1.13	0.58	0.77	0.81	<0.001	0.88	0.32	0.86	0.35	0.486	0.88	0.76	0.89	0.75	0.031	3.17	1.14	2.99	1.17	<0.001
≥$80,000	1.11	0.58	0.88	0.84	<0.001	0.93	0.25	0.91	0.29	0.012	0.97	0.77	1.03	0.76	0.436	3.26	1.17	3.04	1.09	0.162

*M, mean; SD, standard deviation. Score ranges: formal volunteering (0–2), formal monetary nation (0–1), blood and organ donation (0–2), informal helping (0–5). ^a^Altruistic behaviors are four dimensions based on previously validated A-Index.*
